# Visual Properties of Transgenic Rats Harboring the Channelrhodopsin-2 Gene Regulated by the Thy-1.2 Promoter

**DOI:** 10.1371/journal.pone.0007679

**Published:** 2009-11-05

**Authors:** Hiroshi Tomita, Eriko Sugano, Yugo Fukazawa, Hitomi Isago, Yuka Sugiyama, Teru Hiroi, Toru Ishizuka, Hajime Mushiake, Megumi Kato, Masumi Hirabayashi, Ryuichi Shigemoto, Hiromu Yawo, Makoto Tamai

**Affiliations:** 1 International Advanced Interdisciplinary Research, Tohoku University, Sendai, Japan; 2 Department of Medical Biochemistry, Tohoku University Graduate School of Medicine, Sendai, Japan; 3 Department of Developmental Biology and Neuroscience, Tohoku University Graduate School of Life Sciences, Sendai, Japan; 4 Division of Cerebral Structure, National Institute for Physiological Sciences, Okazaki, Japan; 5 Sokendai, Kanagawa, Japan; 6 Solution-Oriented Research for Science and Technology, Kawaguchi, Japan; 7 Section of Mammalian Transgenesis, Center for Genetic Analysis of Behavior, National Institute for Physiological Sciences, Okazaki, Japan; 8 Tohoku University Basic and Translational Research Center for Global Brain Science, Sendai, Japan; 9 Department of Physiology, Tohoku University Graduate School of Medicine, Sendai, Japan; 10 Department of Physiology and Pharmacology, Tohoku University Graduate School of Medicine, Sendai, Japan; Lund University, Sweden

## Abstract

Channelrhodopsin-2 (ChR2), one of the archea-type rhodopsins from green algae, is a potentially useful optogenetic tool for restoring vision in patients with photoreceptor degeneration, such as retinitis pigmentosa. If the ChR2 gene is transferred to retinal ganglion cells (RGCs), which send visual information to the brain, the RGCs may be repurposed to act as photoreceptors. In this study, by using a transgenic rat expressing ChR2 specifically in the RGCs under the regulation of a Thy-1.2 promoter, we tested the possibility that direct photoactivation of RGCs could restore effective vision. Although the contrast sensitivities of the optomotor responses of transgenic rats were similar to those observed in the wild-type rats, they were enhanced for visual stimuli of low-spatial frequency after the degeneration of native photoreceptors. This result suggests that the visual signals derived from the ChR2-expressing RGCs were reinterpreted by the brain to form behavior-related vision.

## Introduction

Retinitis pigmentosa (RP) is a genetically heterogeneous disease characterized by degeneration of the retinal photoreceptor cells. A number of genes responsible for RP have been identified, most of them related to the phototransduction pathways. Patients who have such mutations experience night blindness, loss of their peripheral visual field, and loss of central vision [1]. Although the photoreceptor cells are degenerated in the eyes of RP patients with vision loss, other retinal neurons, including retinal ganglion cells (RGCs), are still preserved [2], [3], [4].

Channelrhodopsin-2 (ChR2), a rhodopsin identified in the green algae Chlamydomonas reinhardtii, is unique in that it acts as a directly light-gated cation-selective ion channel [5]. Several studies have revealed that neurons became photosensitive when transfected with the ChR2 gene [6], [7]. In addition, Bi et al. reported that the transfer of ChR2 restored visually evoked cortical responses in blind mice [8]. We also observed restoration of visual response in genetically blind rats [9]. Following on the study of Bi et al. and our own research, we believe that, in addition to their native function of transmitting visual signals to the brain, RGCs are endowed with a photoreceptor-like function by the ChR2 gene. There are three types of RGCs in the mammalian retina: ON, OFF, and ON-OFF [10], [11]. Since the transfer of the ChR2 gene into RGCs was not regulated according to RGC type in these studies, it is possible that all RGC types became photosensitive. Thus, RGC-derived signals must be reinterpreted by the brain in order to organize effective vision. Transgenic rats that express ChR2 in RGCs provide a useful experimental model with which to evaluate quantitatively the visual function of an animal in which RGCs are made photosensitive by the expression of ChR2.

The Thy-1.2 antigen is a glycoprotein found on the cell surface of a variety of cell types [12], [13]. Rat Thy-1.2 antigen has been found to be abundant in the brain and thymus [14], [15]. In the retina, the Thy-1.2 antigen is recognized to be a marker specific to RGCs [16], [17]. Thus, the Thy-1.2 promoter is an effective regulator of a gene that is expressed exclusively in the RGCs [18], [19], [20]. In the present study, we generated transgenic rats in which the ChR2 transgene was driven by the Thy-1.2 promoter. One of them, line 4 (W-TChR2V4), expressed ChR2 specifically in the RGCs of the entire retina. We found that contrast sensitivities of optomotor responses in W-TChR2V4 rats were equivalent to wild-type rats, even when native photoreceptor cells were degenerated by continuous light exposure. However, contrast sensitivities at low spatial frequencies were enhanced after photoreceptor cell degeneration. This suggests that the visual signals derived from the ChR2-expressing RGCs are reinterpreted to form behavior-related vision.

## Results

### Generation of Transgenic Rats

**Figure 1:**
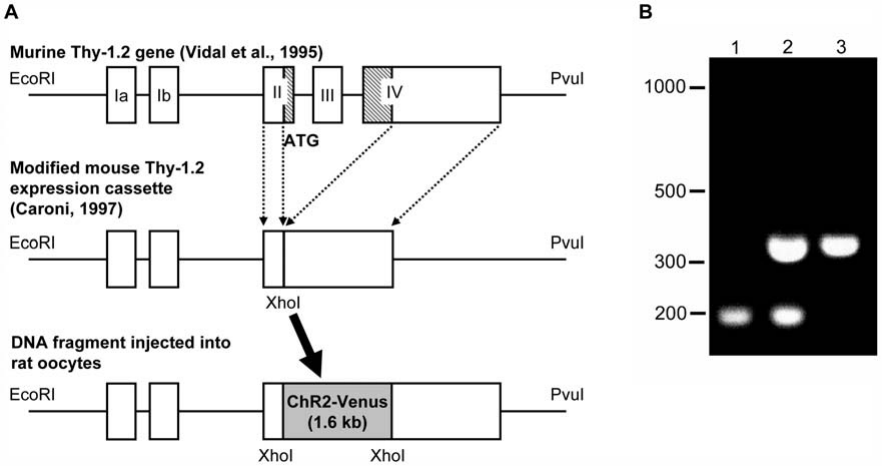
Generation of Thy-1.2 ChR2V transgenic rat. Schematic drawing of DNA fragment injected into rat oocytes. (A) The cDNA coding channelrhodopsin-2 (ChR2) tagged with Venus was inserted at XhoI site of the modified mouse Thy-1.2 expression cassette. A linearized DNA fragment (7.5 kb) prepared by digestion with EcoRI and PvuI restriction enzymes was injected into rat oocytes. (B) Examples of PCR analysis of genomic DNA from transgenic founder rats injected with the transgene shown in A. Genomic DNAs from the injected DNA fragment (lane 1), a transgenic founder (lane 2) and a non transgenic founder (lane 3) were amplified by PCR. DNA bands at 173 bp and 324 bp correspond to amplified DNA fragments for the transgene (ChR2-Venus, ChR2V) and the T cell receptor gene as an internal control, respectively.

The Thy-1.2 vector derived from a 6.5-kb fragment of the murine Thy-1.2 gene has been reported to promote gene expression in RGCs and in neurons in the brain [21] (Fig. 1A). We analyzed the genomic insertion of a ChR2V cDNA fragment by performing polymerase chain reaction (PCR) on tail DNA and subsequently detected a PCR product of 324 bp in eight founder rats (Fig. 1B). We termed these transgene positive lines “Wistar-Thy-1.2 promoter-Channelrhodopsin 2-Venus rats” (W-TChR2V). Among these 8 lines (W-TChR2V1-8), 6 lines, which were capable of reproduction and transgenerational propagation of the transgene, were evaluated further for expression of the ChR2V protein in the retina.

**Figure 2:**
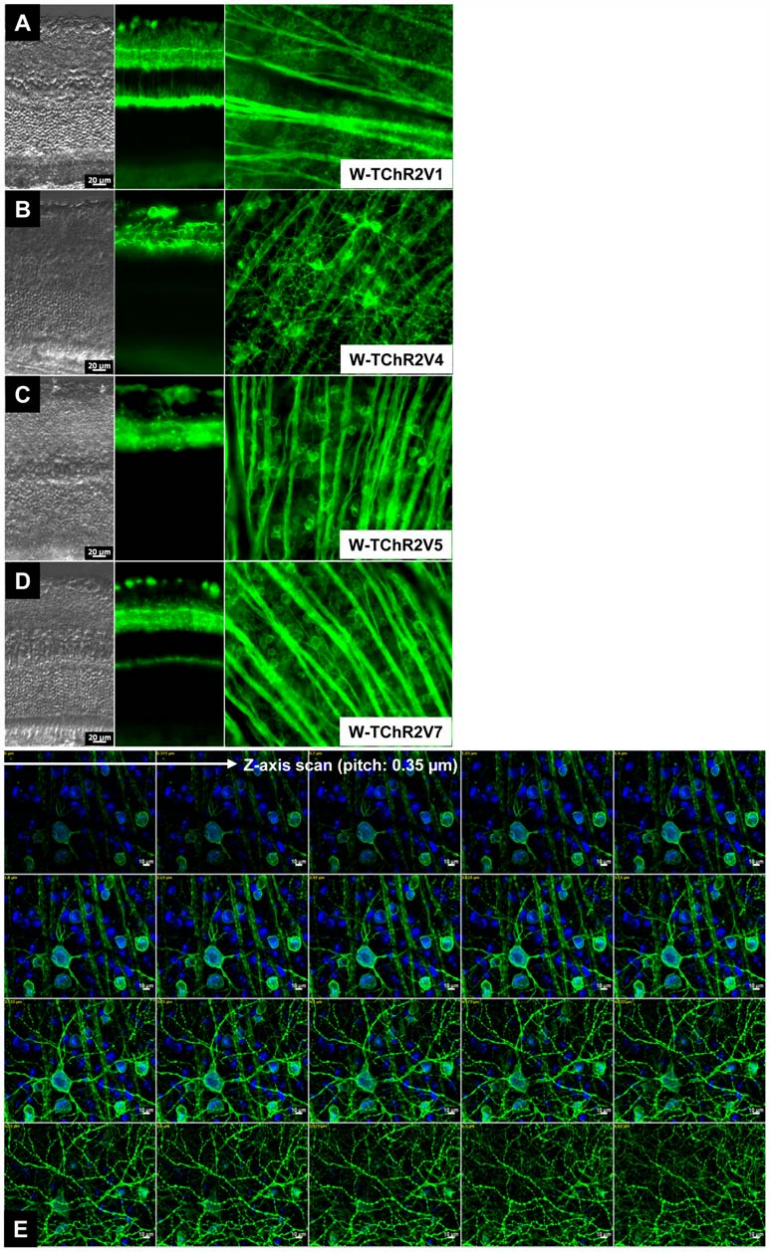
Microphotographs showing ChR2V expression in the inner retinal layers of each transgenic line. (A–D) The retinal organization of each transgenic line (A, W-TChR2V1; B, W-TChR2V4; C, W-TChR2V5; D, W-TChR2V7) showed normal features in the Nomarskii images (left). Fluorescence microphotography revealed various expression patterns in retinal slices (middle) and flat-mounted retinas (right). (E) Z-axis scan (pitch: 0.35 µm) images collected from a flat-mounted retina of a ChR2V+/− rat (line W-TChR2V4) showed that the ChR2V fluorescence (green) was coexpressed with fluorogold transported retrograde from the superior colliculus (blue).

Under fluorescence microscopy, ChR2V was shown to be expressed in the retina of the heterozygous rat (ChR2V +/−) in four of six lines of transgenic rats: W-TChR2V1, W-TChR2V4, W-TChR2V5, and W-TChR2V7 (Fig. 2A–D). As shown in Fig. 2, ChR2V expression was extensively observed in the flat-mounted retina. Vertical sections indicated that cells expressing ChR2V were distributed differently in each transgenic line. In the case of W-TChR2V1, the “Venus” marker fluorescence (see Methods section) was observed in the RGC layer (GCL), inner plexiform layer (IPL), and outer plexiform layer (OPL). The W-TChR2V4 strain showed ChR2V expression in the GCL and IPL. In addition to expression in these layers, strains W-TChR2V5 and W-TChR2V7 showed intense fluorescence in the inner nuclear layer (INL). When the flat-mounted retina of the W-TChR2V4 rat was vertically examined using the z-axis scanning mode of the microscope, the Venus fluorescence was colocalized with Fluorogold, which retrogradely labeled the RGCs (Fig. 2E).

### Direct Photoactivation of ChR2V-Expressing RGCs

**Figure 3:**
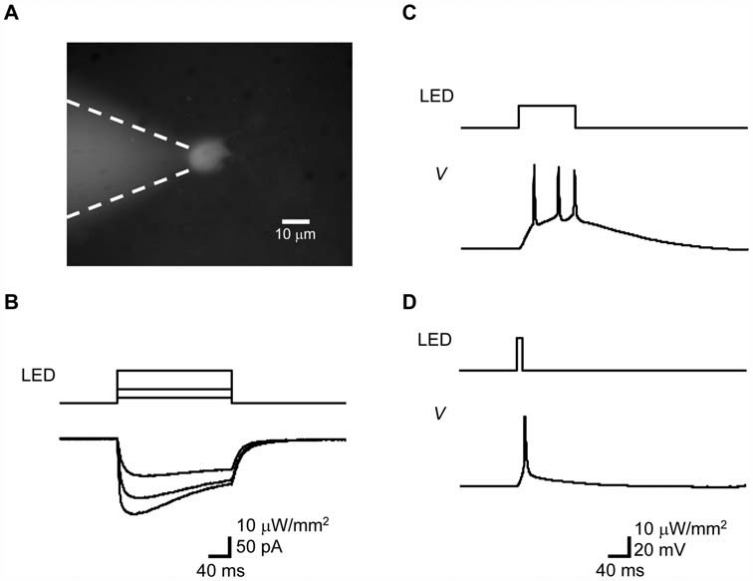
Direct photoactivation of ChR2V-expressing RGCs. (A) The blue light-emitting diode (LED)-evoked membrane currents and potentials were recorded under whole-cell recording from one of the ChR2V-expressing RGCs. (B) The photocurrents and their dependency in the LED power density. (C–D) The membrane potential responses of a ChR2V-expressing RGC to LED pulses of 100-ms (C) or 10-ms (D) duration.

We expected that the ChR2V-expressing RGCs in the W-TChR2V4 rat retina would be sensitive to light. To test this hypothesis, we investigated the light-evoked responses of ChR2V-expressing RGCs, while all the synaptic inputs derived from the photoreceptor cells were pharmacologically blocked by 1 mM kynurenic acid, a nonselective glutamate receptor blocker. In a ChR2V-expressing RGC placed under whole-cell voltage clamp at −60 mV (Fig. 3A), a blue light-emitting diode (LED) light pulse evoked an inward current whose amplitude was dependent on the light power density (Fig. 3B). The light-evoked current has similarities to a ChR2 photocurrent [6], i.e. rapid onset without detectable latency, peak-and-plateau biphasic kinetics, and a rapid offset. The onset time constant was dependent on the light power density, but 4–9 ms in this case. The offset time constant was less dependent on the light power density and was 15–18 ms. Under current-clamp configuration, membrane potential was depolarized by an LED light pulse with an undetectable delay and was accompanied by action potentials (Fig. 3C). Action potentials were evoked by a 100-ms LED pulse with a power density as low as 3.7±2.3 µW/mm^2^ (n = 14). We found that the action potential could also be evoked by an LED light pulse as short as 10 ms (Fig. 3D).

### Degeneration of Photoreceptor Cells

**Figure 4:**
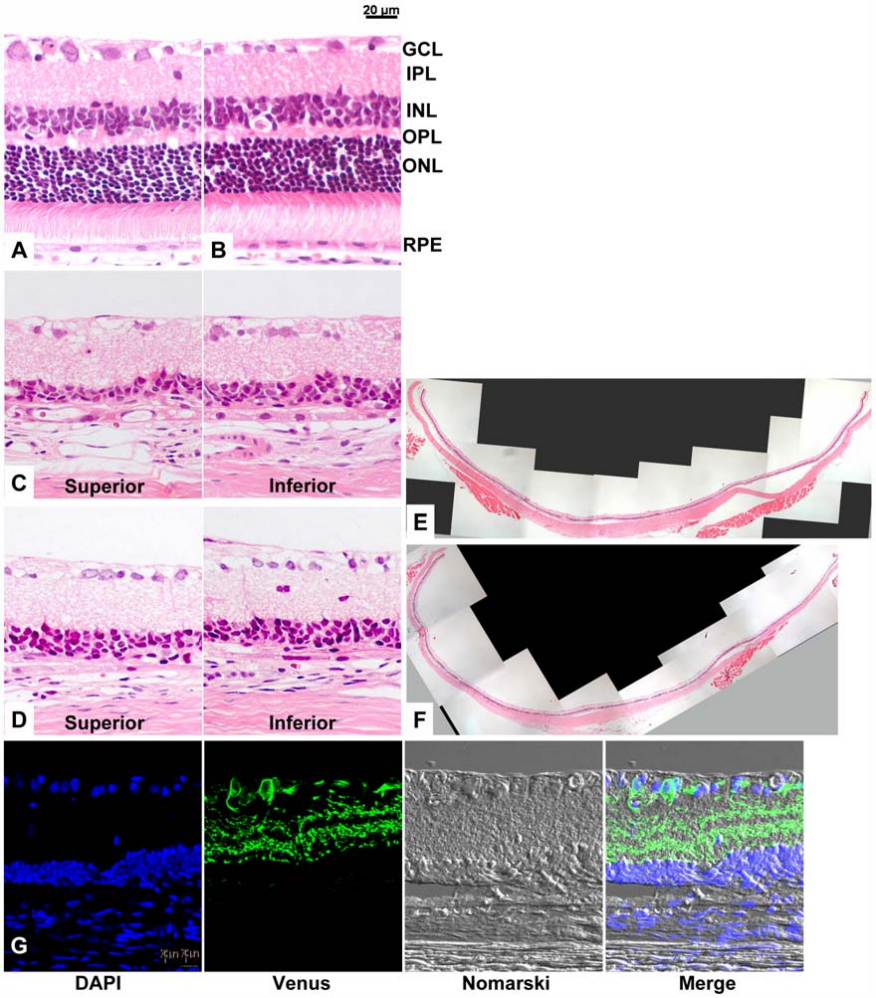
Morphological evidences of the photoreceptor degeneration in either ChR2V−/− or ChR2V+/− rats. (A, B) Normal retinal architecture was observed in each rat before photoreceptor degeneration. (C, D) After continuous light exposure (3000 lux for 7 days). Sections are from the superior and inferior regions at a distance of 0.24 mm from the optic nerve. Note the absence of the outer nuclear layer. (E, F)The severe degeneration extended to the whole retina. (G)Cryo-section of a retina from a ChR2V+/− rat. ChR2V expression was observed in the inner layer. Abbreviations: GCL, ganglion cell layer; IPL, inner plexiform layer; INL, inner nuclear layer; OPL, outer plexiform layer; ONL, outer nuclear layer; RPE, retinal pigment epithelium.

There were 11–12 rows of photoreceptor nuclei in the outer nuclear layer (ONL) of the transgenic rats; this is a number usually observed in rodents without retinal degeneration [22] and indicates that the ectopic expression of ChR2V did not affect retinal structure (Fig. 4A, B). After continuous toxic light exposure, the cells in the ONL were almost absent in both the superior and inferior retinas from both the ChR2V−/− (Fig. 4C) and ChR2V+/− rats (Fig. 4D). The disappearance of the ONL was also noted under low magnification in a slice of the whole retina (Fig. 4E, F). Even after photoreceptor degeneration, the ChR2-expressing RGCs remained in the ChR2V+/− rats (Fig. 4G).

**Figure 5:**
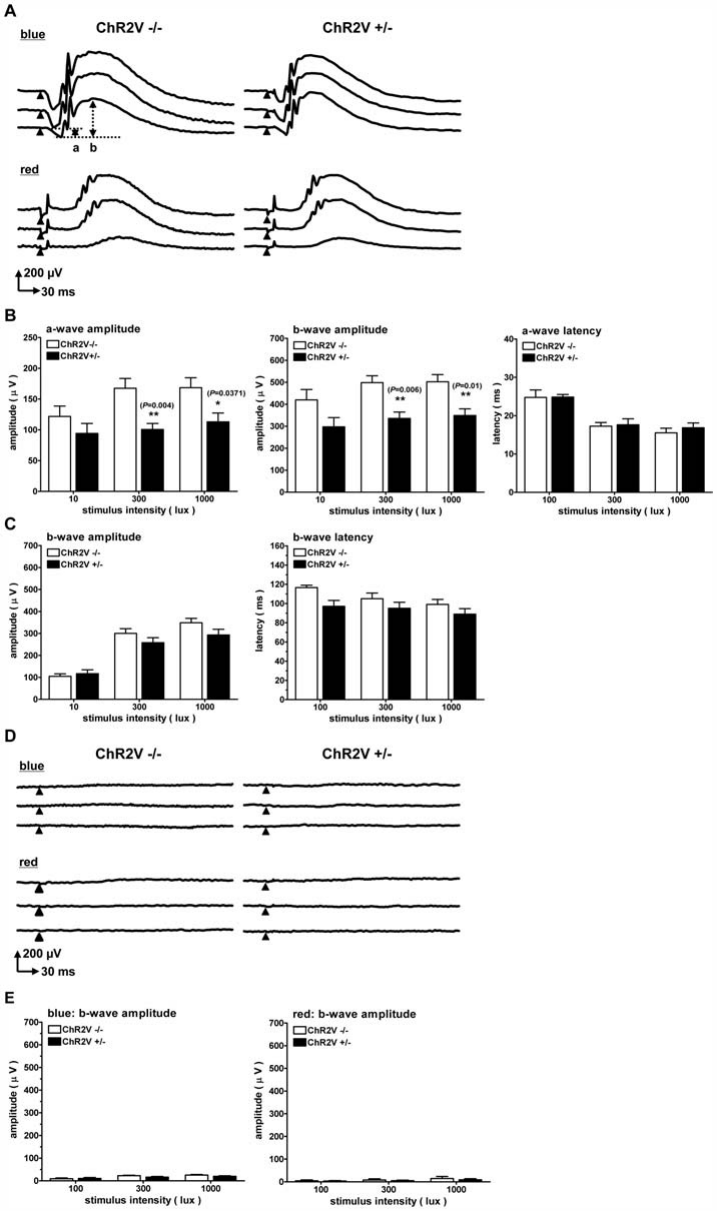
Electrophysiological evidence of photoreceptor degeneration. (A) Typical waveforms of electroretinogram (ERG) responses evoked by either blue or red light-emitting diode (LED) flash (duration: 10 ms; light intensity: 1000, 300, and 100 lux, top to bottom). (B)The ERG amplitudes (left, a-wave; middle, b-wave) and the latency of the a-wave (right) in response to the blue LED flash. Note that both amplitudes were significantly diminished in the ChR2V+/− rats compared to the ChR2V−/− rats without any differences in a-wave latency. (C) The b-wave amplitudes (left) and the latency (right) in response to the red LED flash. (D)Typical ERG waveforms evoked by either blue (upper traces) or red (lower traces) LED flash in the ChR2V−/− (left) and +/− (right) rats after continuous light (3000 lux) exposure for 7 days. (E)The b-wave amplitudes recorded from the rats after continuous light exposure. Error bars represent standard deviation (n = 8, **: P<0.01, unpaired t-test).

When the visual signals generated by the photoreceptor cells are transmitted to inner retinal neurons, the associated change in the electric field of the retina is evaluated as the electroretinogram (ERG) response. Typical waveforms of ERGs were observed in either the ChR2V−/− or the ChR2V+/− rats (Fig. 5A; upper). We found that the a- and b-wave ERG amplitude was small in the ChR2V+/− rats when evoked by the blue LED light. Both a- and b- wave amplitudes were significantly higher in ChR2V−/− than in ChR2V+/− rats (Fig. 5B). With regard to the latency of the a-wave, no detectable difference was observed between the ChR2V−/− and ChR2V+/− rats (Fig. 5B). On the other hand, the ERG response of the ChR2V+/− rats was quantitatively similar to that of the ChR2V−/− rats when evoked by the red LED light (Fig. 5A; lower, C). Since the blue LED light, but not the red LED light, was absorbed by both the ChR2 and Venus protein, photon density of the blue LED light may have been reduced before reaching the photoreceptor cells.

After exposing the rats to 3000-lux light continuously for 7 days, the ERG responses were evaluated with the blue LED and red LED (Fig. 5D). The ERG responses were almost negligible with either the blue or the red LED at intensities of 10–1000 lux. Amplitudes of the a- or b-wave were markedly decreased in both the ChR2v −/− and +/− rats, which indicated that retinal function had been damaged by the continuous light exposure (Fig. 5E). This ERG reduction was adopted as a criterion of photoreceptor degeneration in the following experiments.

### Visually Evoked Potentials

**Figure 6:**
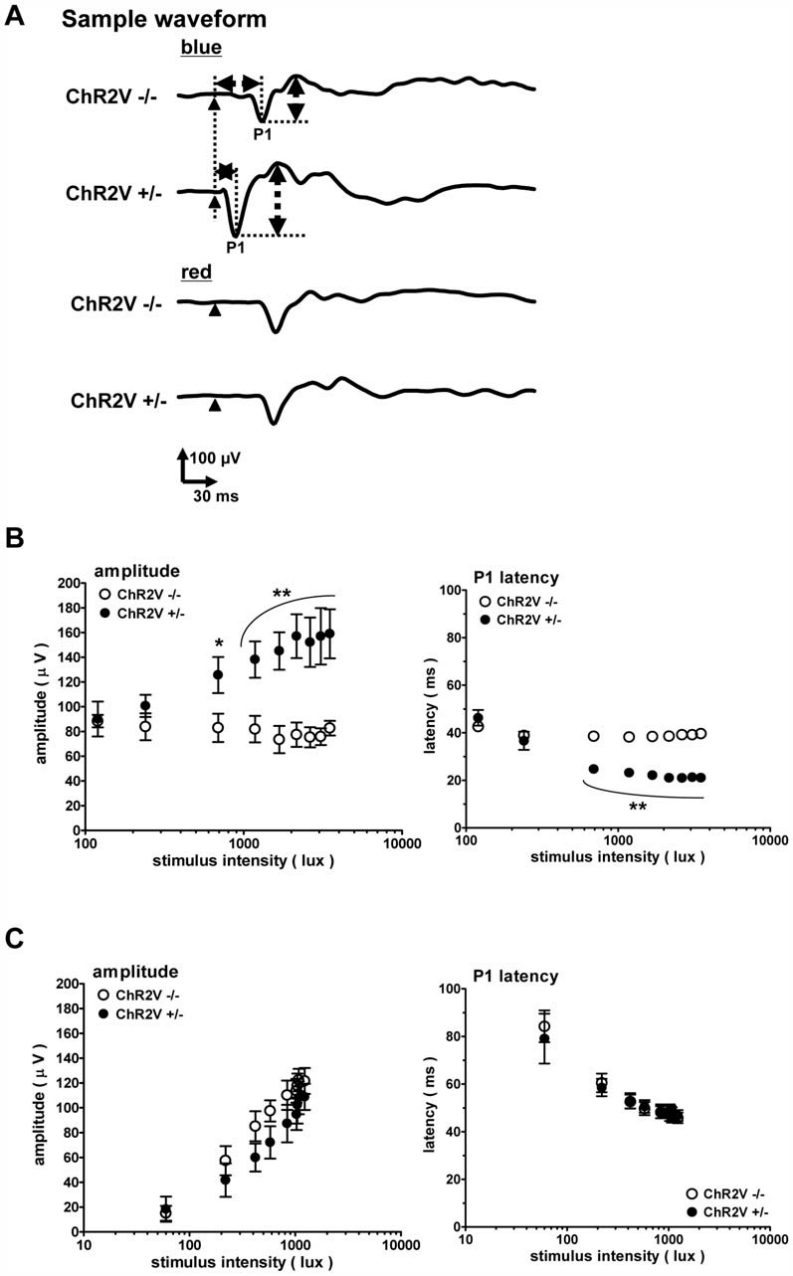
The visually evoked potentials recorded from either the ChR2V−/− or ChR2V+/− rats before photoreceptor degeneration. (A)Sample waveforms evoked by the blue or red LED flash. (B) The amplitude- (left) and the latency- (right) stimulus intensity relationships of VEPs evoked by the blue LED flash. (C) Similar to (A), but the responses are to the red LED flashes. Error bars represent standard deviation (n = 8, *: P = 0.04, **: P<0.01, unpaired t-test).

In a normal eye, the visual signal is first received by the photoreceptor cells, transmitted and integrated in the retinal neuronal network, projected to the brain by the RGCs, eventually arriving at the visual cortex through synapses in the lateral geniculate nucleus. This signaling chain is evaluated as a whole by the visually evoked potential (VEP), a visual cortical response triggered by a short light pulse. Figure 6A shows sample rat VEPs before inducing photoreceptor degeneration. VEPs were recorded in both the ChR2V−/− and ChR2V+/− rats. When the VEPs were evoked by the weak blue LED light, those of the ChR2V+/− rats were similar to those of the ChR2V−/− rats. However, with the strong blue LED light (>240 lux), the VEP of the ChR2V+/− rat was larger in amplitude and shorter in latency than that of the ChR2V−/− rat (Fig. 6B). This suggests that the strong blue LED light induces the ChR2V-expressing RGCs to fire directly, without mediation by photoreceptor cells.

On the other hand, when VEPs were evoked by the red LED light, those of the ChR2V+/− rats were similar to those of the ChR2V−/− rats in both amplitude and time course (Fig. 6A). No significant differences were present in the VEP amplitude-stimulus intensity relationships, and the latency-stimulus intensity relationship of the ChR2V+/− rats was also identical to that of the ChR2−/− rats (Fig. 6C).

**Figure 7:**
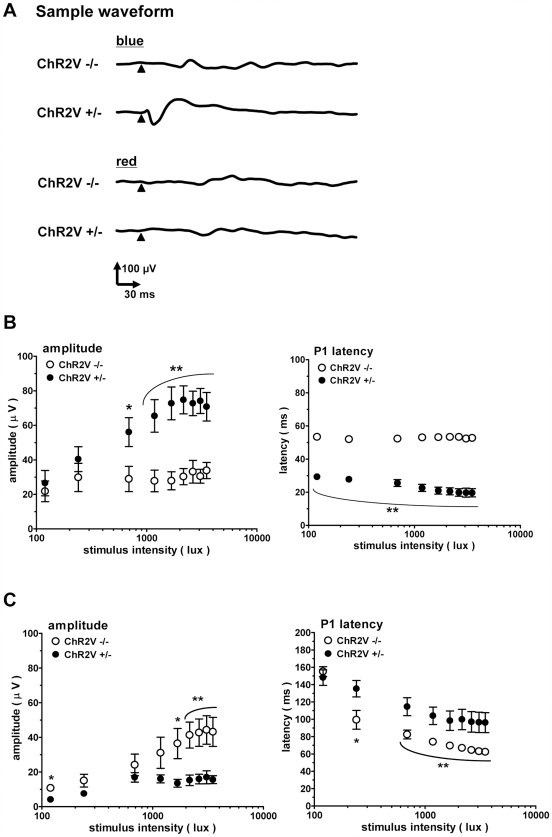
The VEPs after photoreceptor degeneration. (A)Sample waveforms evoked by blue or red LED flash. (B) The amplitude- (left) and the latency- (right) stimulus intensity relationships of VEPs evoked by the blue LED flash. (C) The summary of VEP responses to the red LED flashes. Error bars represent standard deviation (n = 8, *: P<0.05, **: P<0.01, unpaired t-test).

The VEPs were then recorded after exposing the rats to 3000 lux light continuously for 7 days. After subsequent degeneration of most of the photoreceptor cells, the VEPs resulting from either blue or red light were almost negligible in the ChR2V−/− rats (Fig. 7A). In contrast, in the ChR2V+/− rats, the VEPs evoked by the blue LED light clearly remained, even after photoreceptor degeneration (Fig. 7B); however, the VEPs evoked by the red LED were negligible (Fig. 7C). As shown in the amplitude-stimulus intensity relationship (Fig. 7B), the remaining VEPs were induced only by strong blue LED light (>240 lux). These VEPs were again characterized by shortened latency periods.

### Optomotor Responses of the Rats

**Figure 8:**
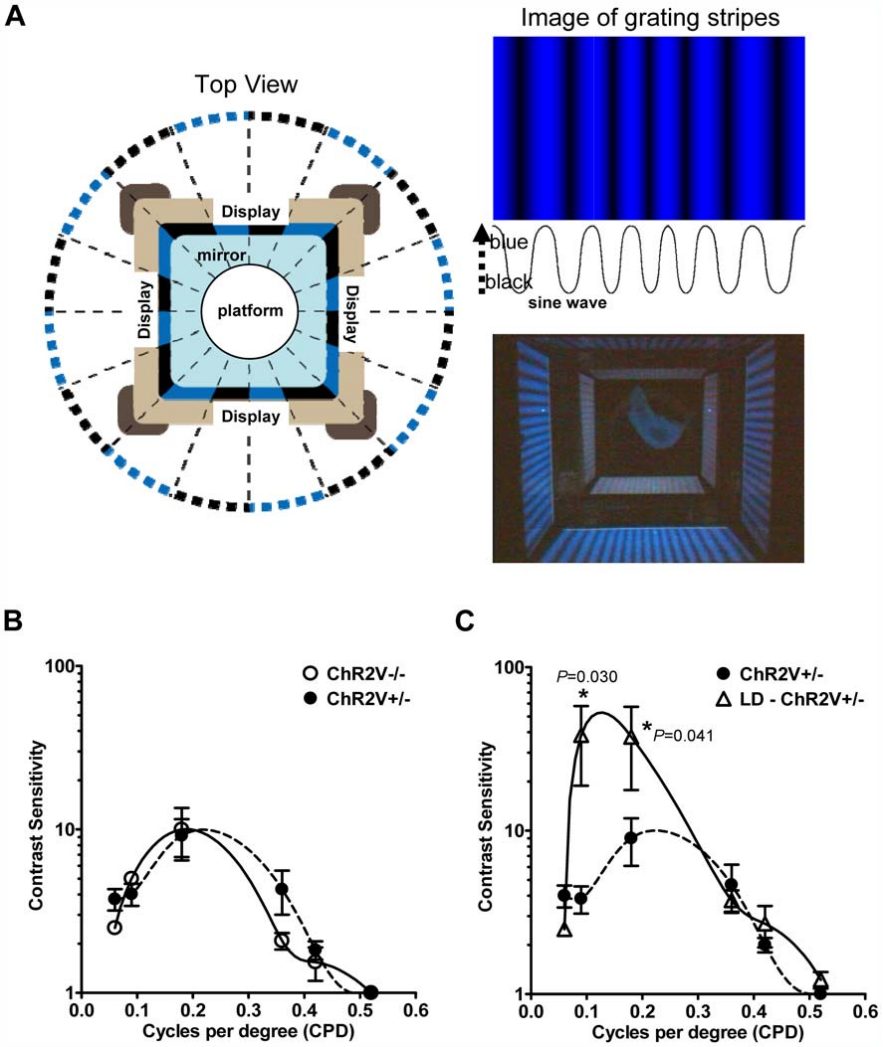
Optomotor response of the ChR2V−/− and ChR2V+/− rats. (A) The experimental design for the evaluation of optomotor response. The moving vertical stripes were displayed on the computer monitors so that the brightness-darkness contrast followed a sine-wave function of variable amplitude and spatial frequency. (B)The contrast sensitivity-spatial frequency relationship of the ChR2V−/− and ChR2V+/− rats before photoreceptor degeneration. (C) The contrast sensitivity-spatial frequency relationship of the ChR2V+/− rats after photoreceptor degeneration. LD  =  Light damaged. Error bars represent standard error of the mean (n = 8, *: P<0.05, Mann-Whitney U-test).

The spatial vision of an animal was quantified by its optomotor response. When a drum is rotated around an animal with printed visual stimuli on the inside wall, the animal tracks the stimulus by turning its head [23]. In our virtual optomotor system, a stimulus of blue stripes over a black background was produced according to a sine wave function with variable amplitude and frequency (Fig. 8A). With a given spatial frequency, the rat tracked the objects if the brightness-darkness contrast was high. However, the rat's response became undetectable when this contrast was reduced. We changed the blue/black contrast while keeping the mean brightness on the platform constant at 100 lux.

The Michelson contrast at a given spatial frequency is evaluated as follows:
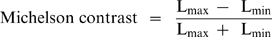
where L_max_ is the maximal brightness (in lux) of the stimulus measured on the stage, and L_min_ is the minimal brightness (in lux). Each animal in this study tracked the virtual rotating blue/black gratings at the maximal contrast of 1. However, it stopped tracking when the contrast was reduced below a specific threshold. The reciprocal of this threshold was defined as the contrast sensitivity; once calculated, its dependence on the spatial frequency was investigated (Fig. 8B). We observed that contrast sensitivity was small at the minimal spatial frequency of 0.06 cycles per degree (CPD), increased with the increase of spatial frequency, reached a maximum around 0.18 CPD, and was negligible at spatial frequencies over 0.52 CPD. Therefore, the relationship followed an inverted U-shaped curve, as noted in previous reports [23], [24]. We found that the optomotor responses of the ChR2V+/− rats were similar to those of the ChR2V−/− rats with regard to the contrast sensitivity-spatial frequency relationship.

The optomotor response of ChR2V−/− rats was severely reduced after exposing the rats to 3000-lux light continuously for 7 days. They no longer tracked the virtual rotating blue/black gratings at any spatial frequency, even with a maximal contrast of 1. In the ChR2V+/− rats, no reduction of contrast sensitivity was observed at any spatial frequency, even after photoreceptor degeneration (Fig. 8C and Movie S1). Unexpectedly, the contrast sensitivity was instead somewhat enhanced at a low spatial frequency such as 0.09 or 0.18 CPD.

## Discussion

In the present paper, we tested the possibility that RGCs can behave as photoreceptor cells when they are endowed with photosensitivity. We investigated this by using a line of transgenic rats, W-TChR2V4, in which the ChR2 gene was expressed selectively in RGCs under the regulation of the Thy-1.2 promoter. In heterozygous rats where ChR2 was conjugated with a fluorescent marker, ChR2 was exclusively expressed in the RGCs. Stimulation with blue LED light directly evoked action potentials in the ChR2V-expressing RGCs. The VEP in transgenic rats with intact photoreceptors consisted of two components with either a short or a long latency. The short-latency component appeared to be derived from the direct photosensitive response of the RGCs, whereas the long-latency component was dependent on the retinal rod photoreceptor cells. The head-tracking behavior of the ChR2V+/− rats showed that they followed the virtual rotation of light-dark gratings even after the massive degeneration of photoreceptor cells. This evidence suggests that ChR2-expressing RGCs are substitutable for rod photoreceptor cells in the retina.

### Selective Expression of ChR2 in the RGCs

In the present study, we established several lines of transgenic rats in which ChR2 expression was driven by the Thy-1.2 promoter. The phenotypic expression of ChR2 was detected in the retina by the coexpression of the fluorescent protein “Venus,” which is connected at the C-terminal end of the N-terminal 315 amino acid fragment of ChR2. We found that phenotypic expression varied from line to line. In some lines such as W-TChR2V1, W-TChR2V5, and W-TChR2V6, ChR2 was expressed in nonganglion cells such as amacrine, bipolar, and Müller. This is consistent with findings that the Thy-1.2 antigen is expressed in some of these cells at low level, even though Thy-1.2 has been considered to be a “specific” marker of RCGs [16], [17], [25]. Feng et al. [26] observed various GFP expression patterns among transgenic mouse lines in which GFP expression was driven by the Thy-1.2 gene. This variation may reflect differences in integration sites in the chromosomes and/or the number of inserted copies. One of our lines, W-TChR2V4, expressed ChR2 nearly selectively in RGCs. Therefore, this line appeared to be the most suitable line for studying visual function produced by ChR2-transferred RGCs. When the RGCs of this line were retrogradely labeled with a fluorogold applied to the superior colliculus, 45.7%±8.4% of the fluorogold-positive RGCs were also expressing ChR2V. It is possible that ChR2V-positive and –negative RGCs have different morphological and/or physiological traits [10], [11], [27], [28], [29], [30], although further experiments are necessary to reveal such correlations.

### Visual Responses Dependent on the ChR2-Expressing RCGs

One of our novel findings was the presence of an early VEP component in the ChR2V+/− rats. The mean latency of this component was 20 ms, suggesting that no more than one synapse was involved in the pathway. The early VEP component was only evoked by bright blue LED light of over 240 lux, but was not evoked by the red LED light. This is consistent with the notion that this VEP response was derived from the direct and rapid depolarization of the ChR2-expressing RGCs, since ChR2 is exclusively sensitive to the blue light, with a peak sensitivity at 460–480 nm [5]. After photoreceptor degeneration, the early VEP component remained in the ChR2V+/− rats, while the late component was almost extinguished. This evidence strongly suggests that the blue LED light directly evoked action potentials in these neurons and that the signal was conducted to the visual cortex via synapses in the lateral geniculate nucleus. That is, the ChR2-expressing RGCs acted as extra photoreceptors in these animals. Thus, the visual cortex accepted the signals derived from these photosensitive RGCs in parallel with those derived from retinal photoreceptor cells. Previously, we reported that the VEP was restored when the ChR2 gene was delivered to the retinal cells in aged dystrophic Royal College of Surgeons (RCS) rats in which the photoreceptor cells were degenerated [9]. Re-evaluation of this VEP latency revealed that it was approximately 20 ms. This was smaller than the latency of the VEP evoked in nondystrophic RCS rats without ChR2 gene induction.

We also investigated the optomotor response of the ChR2V+/− rats using virtual rotating blue/black gratings. The head-tracking behavior with contrast sensitivity in these animals was no less than that of the ChR2V−/− rats. Thus, the signals from the ChR2-expressing RGCs appear not to have interfered with those derived from the photoreceptor cells. Therefore, it is possible that these signals are integrated in the visual cortex to produce appropriate vision.

### Visual Responses after Photoreceptor Degeneration

To explore the visual function produced by ChR2-expressing RGCs, we induced the degeneration of native photoreceptor cells using the light-induced photoreceptor degeneration model [31], which is commonly used to study the mechanisms of stress-induced photoreceptor degeneration [32], [33], [34]. In this animal model as well as in inherited retinal degeneration, the “final common pathway” of photoreceptor cell death is considered to be apoptosis [35], [36] although cones in retinal degeneration (RD), even if they loose their function, can survive for very long time, i.e. cone apoptosis does not occur necessarily in some RD patients [37]. In the present study, rats were exposed to 3000 lux light continuously for 7 days so that photoreceptor cells were not protected by the mechanisms underlying bright cyclic light rearing [38], [39], [40]. When the retinas of the light-exposed animals were histologically examined, the photoreceptor cells had almost disappeared. The ERG responses were almost entirely extinguished in the present study; therefore, the native photoreceptor activity was clearly diminished by the continuous light exposure.

Even in the absence of photoreceptor cell activity, the visual cortex of the ChR2V+/− rats received visual signals derived from the ChR2-expressing RGCs, as evidenced by the remaining early VEP component. Are these signals correctly interpreted by the brain to form behavior-related vision? In the present study, we investigated the optomotor response of the ChR2V+/− rats after continuous light exposure. These animals did indeed track the virtual rotating blue/black gratings with contrast sensitivity no less than before photoreceptor degeneration. Thus, we suggest that the visual signals derived from the ChR2-expressing RGCs are reinterpreted by the brain as some form of vision.

Nevertheless, these animals did appear to sense their environment differently from the controls. For example, they were only sensitive to bright blue light. With this light stimulus, they showed enhanced contrast sensitivity to the virtual rotating gratings at low spatial frequency. It is possible that the visual signals derived from the ChR2-expressing RGCs are particularly suitable to this kind of visual information. On the other hand, the visual signals derived from the native photoreceptor cells appear to be adapted to a broader range of spatial frequencies. Although these two pathways may possibly raise a visual rivalry in the visual system, the pathway driven by the photoreceptor cell activation may overcome the pathway driven by activation of the ChR2-expressing RGC. Further studies are necessary to evaluate this possibility.

In conclusion, we created a model transgenic rat system and demonstrated that RGCs behave as additional photoreceptor cells if they are expressing ChR2. Since RGCs are preserved in the retina of patients with photoreceptor degeneration, such as that occurring in retinitis pigmentosa, the delivery of the ChR2 gene would restore patients' vision to some extent. Since RGCs are physiologically heterogeneous [28], [41], it would be ideal if ChR2 were expressed exclusively in the ON-type RGCs for improvement of vision. Our study revealed that the visual signals derived from the ChR2-expressing RGCs are reinterpreted by the brain to form a kind of vision, even if the expression is nonselective.

## Materials and Methods

### Animals

All experiments were conducted with the approval of the Animal Research Committee, Graduate School of Medicine, Tohoku University and the National Institute for Physiological Science's Animal Care and Use Committee. Rats were kept in cyclic light (12 hours ON/OFF: 200 lux/dark) after birth and fed laboratory chow *ad libitum* with free access to water.

### Generation of Transgenic Rats

We followed the protocol previously described by Feng et al. [26] to generate Thy-1.2 transgenic rats. The Thy-1.2 vector was generously provided to us by Dr. Joshua Sanes (Washington University, Saint Louis, MO) and has been described by Vidal et al. [42], Kelley et al. [43], and Caroni et al. [21] (Fig. 1A). The Thy-1.2 vector contained 6.5 kb of the murine Thy-1.2 gene extending from the promoter to the intron following exon 4, without exon 3 and the flanking introns [21]. The targeting vector was constructed by inserting a DNA fragment coding the ChR2 (a generous gift from Dr. G. Nagel, Universität Würzberg, Würzberg, Germany) fused to the Venus gene (a generous gift from Dr. A. Miyawaki, RIKEN BSI, Wako-shi, Japan) into the XhoI site of the Thy-1.2 vector (Thy-1.2-ChR2V; Fig. 1A). A 8.1-kbp of Thy-1.2 ChR2V DNA solution at a concentration of 5 µg/ml was microinjected into pronuclear-stage zygotes of Wistar rats to produce transgenic rats [44]. Transgenic founders were crossed for one to four generations before initiating a detailed analysis of expression patterns.

### Screenings of Transgenic Line

Rats were screened by genomic PCR for the presence of the transgene. Genomic DNA was isolated by incubating rat tail (−3 mm) in 500 µl of tail lysis buffer (100 mM Tris-HCl [pH 8.0], 5 mM EDTA [pH 8.0], 200 mM NaCl, 0.2% [w/v] SDS, and proteinase K 100 µg/ml) overnight at 55°C. The mixture was shaken vigorously and centrifuged at 12000 rpm for 10 min at room temperature. Five hundred µl of isopropanol was added to the supernatant, and the contents mixed by inversion. The stringy precipitate of DNA was transferred to a new tube with a clean glass capillary. The DNA was dissolved in 300 µl of TE (pH 8.0). The forward primer (5′-TCTGAGTGGCAAAGGACCTTAGG-3′) and reverse primer (5′-CGCTGAACTTGTGGCCGTTTACG-3′) for the cDNA sequence of fluorescent protein were used at an annealing temperature of 62°C. A primer pair for the T cell receptor gene (5′-CAAATGTTGCTTGTCTGTG-3′ as a forward primer and 5′-GTCAGTCGAGTGCACAGTTT-3′ as a reverse primer) was used for positive control of the genomic DNA. For the examination of the expression of ChR2-Venus in the retina, a few rats of each positive line were perfused with a fixative solution containing 4% paraformaldehyde and 15% saturated picric acid in 0.1 M phosphate buffer (pH 7.2) under deep anesthesia. Eyes were removed and fixed further with 4% paraformaldehyde in 0.1 M phosphate buffered saline (PBS) overnight at 4°C. The flat-mounted retina was made with one of the pair of eyes. The contralateral eyes were embedded in optimal cutting temperature (OCT) compound (Sakura, Tokyo, Japan) following immersion in 30% sucrose solution with PBS. Ten-micrometer retinal sections were made and mounted on slides. The flat-mounted retinas and sections were covered with Vectashield medium (Vector Laboratories, Burlingame, CA). For staining of nuclei with DAPI, retinal slices were covered with Vectashield medium including DAPI (Vector Laboratories). Venus fluorescence was visualized under the Axiovert40 fluorescence microscope (Carl Zeiss).

### Maintenance of Transgenic Rat Lines

Transgenic lines were maintained by cross breeding for more than four generations with the genetic background of Wistar rats. Littermates were screened by genomic PCR using the primers indicated above. Two-month-old littermates were divided to two groups as negative (ChR2V−/−) and positive (ChR2V+/−) for the induction of photoreceptor degeneration.

### Retrograde Labeling of RGCs with a Fluorescent Tracer, Fluorogold

To identify RGCs in the ganglion cell layer (GCL), retrograde labeling was performed 7 days before the rats were sacrificed. The labeling was done by injecting 4 µl of 2% aqueous fluorogold (FG; Fluorochrome, Englewood, CO) [45] containing 1% dimethyl sulfoxide (DMSO) into the superior colliculus using a Hamilton syringe with a 32 G needle [46].

### Induction of Photoreceptor Degeneration

To induce severe photoreceptor degeneration, the conditions under which the rats were kept was changed to cyclic light (12 hours ON/OFF: 5–10 lux/dark) at least 2 weeks before the light exposure. Rats were then exposed to 3000-lux intensity of fluorescent light for 7 days. We used a light exposure box (NK Systems, Tokyo, Japan) to control the timing and light intensity for the induction of photoreceptor degeneration.

### Histological Studies of Retina

Analysis of retinal morphologies in ChR2V−/− and ChR2V+/− rats were performed as previously described by Li et al [32]. In brief, rats were sacrificed by asphyxiation with carbon dioxide after the induction of photoreceptor degeneration. The eyes were enucleated, fixed, and embedded in paraffin. Three-micrometer thick sections of retinas were cut along the vertical meridian and stained with hematoxylin and eosin to allow examination of the retina in the superior and inferior hemispheres [47].

### Electrophysiology of RGCs

Rats were ether-anesthetized, and both left and right eyes were quickly removed and dissected in a cutting solution containing (in mM) 229 mannitol, 3 KCl, 26 NaHCO_3_, 1 H_3_PO_4_, and 7 MgCl_2_, pH 7.4 (4°C) equilibrated with 95% O_2_ and 5% CO_2_ mixed gas. The retina was removed from the pigment epithelium, vitreous side up, and superfused by an artificial cerebrospinal fluid (ACSF) containing (in mM) 114 NaCl, 2.5 KCl, 26 NaHCO_3_, 1 NaH_2_PO_4_, 10 mannitol, 2.5 CaCl_2_, 1.3 MgCl_2_, and 10 glucose (pH 7.4 with 95% O_2_ and 5% CO_2_ mixed gas). To block the photoreceptor-derived inputs, kynurenic acid (1 mM, Sigma-Aldrich) was included in the solutions throughout the experiments. Whole-cell patch-clamp recordings were made from ChR2V-expressing cells visually identified under conventional epifluorescent microscopy (BX50WI, Olympus) equipped with a 60× water objective lens (LUMplanPl/IR60×, Olympus), using a conventional patch clamp system (EPC-7 plus, HEKA and Digidata 1440A, Molecular Devices Co., Sunnyvale, CA). The patch pipette solution contained (in mM): 120 KOH, 100 glutamic acid, 5 HEPES, 2.5 MgCl_2_, 2.5 MgATP, 5 Na_2_EGTA, 1.2 leupeptin (pH 7.4 by KOH). In some experiments, 1% dextran tetramethylrhodamine (Molecular Probes, Eugene, OR, USA) was included to facilitate identification of the recorded cell. Liquid junction potentials were not corrected. After establishing a tight seal, the optical filter set was changed to one equipped with a blue LED (470±25 nm wavelength, LXHL-NB98, Lumileds Lighting Inc., San Jose, CA). Pulsed light was emitted by applying square electrical pulses of 1.5–2.0 V. The light power density was directly measured by a thermopile (MIR-100Q, Mitsubishi Oil Chemicals, Tokyo, Japan). All the experiments were carried out at 27–30°C.

### Recording of ERGs and VEPs

ERGs and VEPs were recorded using a Neuropack (MEB-9102; Nihon Kohden, Tokyo, Japan) according to the methods previously described by Tomita et al. [9]. Briefly, rats were dark-adapted overnight, the pupils were dilated with 1% atropine and 2.5% phenylephrine hydrochloride, and the corneas were anaesthetized with 0.5% propacaine hydrochloride. Small contact lenses with gold wire loops were placed on both corneas, and a silver wire reference electrode was placed subcutaneously between the eyes. Flash light stimuli of 10 ms duration were generated by pulse activation of a blue or white LED. Full-field scotopic ERGs were recorded, band-pass filtered at 0.3–500 Hz, and averaged for five responses at each light intensity. The amplitude of the a- and b-wave was measured when both were clearly detected. For recording VEPs, recording electrodes (silver-silver chloride) were placed epidurally on each side 7 mm behind the bregma and 3 mm lateral of the midline, and a reference electrode was placed epidurally on the midline 12 mm behind the bregma at least 7 days before the experiments. Under ketamine-xylazine anesthesia, the pupils were dilated with 1% atropine and 2.5% phenylephrine hydrochloride. The ground electrode clip was placed on the tail. Photic stimuli of 20-ms duration under various intensities were applied with a frequency of 0.5 Hz. Photic stimuli were generated by pulse activation of a blue LED with light emitting wavelengths of 435–500 nm (peak at 470 nm) or a red LED (580–640 nm, peak at 625 nm). The high and low pass filters were set to 50 kHz and 0.05 kHz, respectively. One hundred consecutive response waveforms were averaged for each VEP measurement.

### Behavioral Tests

We used a virtual optomotor system to evaluate the optomotor responses. The original virtual optomotor system described by Prusky et al. [23] was modified for rats. A light-dark grating pattern was displayed on computer monitors (ProLite E1902WS; Iiyama, Tokyo, Japan) arranged in a square around a platform. A video camera was stationed 50 cm above the platform. The grating patterns, which were determined by a sine wave function with variable spatial frequency and contrast, were produced by a program we developed using the Visual Basic 2007 programming language (Microsoft). The software also controlled the speed of virtual optomotor rotation, which was set at 12 degrees per second (2 rpm) in all experiments. The spatial frequency and the contrast of the grating pattern was varied but the average brightness kept constant. The illuminance at the center of the platform was 200, 100, and 0.5 lux when the color was set to white, blue, or black, respectively. Mirrors covered the platform above and below. From the perspective of the rat, the environment was like a 3-D world surrounded by moving light-dark vertical gratings.

The animal was allowed to move freely on the platform in the virtual optomotor system. The experimenter waited until it stopped moving, and then a homogeneous gray stimulus was projected for 30 s on the monitors before the presentation of each grating session, which was also timed for 30 s. The grating session was started from a low spatial frequency (0.06 cycles/degree) with the maximal contrast. An experimenter assessed whether the animals tracked the rotation by monitoring the head movement and the presented rotating stimulus simultaneously on another display connected to the video camera. If head movement simultaneous with the rotation was evident, the experimenter judged that the animal could discriminate the grating and proceeded to the next grating session. If the movement was ambiguous, the same grating session was presented again. All behavioral tests were double blind and performed during the first few hours of the animals' light cycle (light on at 8 AM).

### Statistical Analysis

Statistical analysis was performed using GraphPad Prism software (GraphPad Software, San Diego, CA). The criterion for statistical significance was P<0.05. The statistical methods used were the unpaired t-test and the Mann-Whitney U-test for the electrophysiological studies and the behavioral studies, respectively.

## Supporting Information

Movie S1 The movie of optomotor response in photoreceptor degenerated ChR2V+/− rat. The ChR2V+/− rat was exposed to 3000-lux intensity of fluorescent light for 7 days to induce photoreceptor degeneration. The responses of ERGs were negligible by either blue or red light (Fig. 7A). The head-tracking behavior was evaluated using a virtual optomotor system. Three digits showed the spatial frequency (cycles per degree) under the left.
(2.90 MB MP4)
